# A comparison of different exercise intensities for improving bone mineral density in postmenopausal women with osteoporosis: A systematic review and meta-analysis

**DOI:** 10.1016/j.bonr.2022.101631

**Published:** 2022-10-21

**Authors:** Takashi Kitagawa, Kaede Hiraya, Takumi Denda, Shuhei Yamamoto

**Affiliations:** a Department of Physical Therapy, School of Health Sciences, Shinshu University, Matsumoto, Japan; b Department of Rehabilitation, Shinshu University Hospital, Matsumoto, Japan

**Keywords:** aBMD, areal bone mineral density, BMD, bone mineral density;, CI, confidence interval, FN, femoral neck, HiRIT, high-intensity resistance and impact training, LS, lumbar spine, MiRIT, moderate-intensity resistance and impact training, RCT, randomized controlled trial, RM, repetition maximum, SMD, standardized mean difference, Bone mineral density, Exercise, Meta-analysis, Osteoporosis, Postmenopausal women, Resistance training

## Abstract

**Objective:**

This study aimed to compare the effects of moderate- and high-intensity resistance and impact training (MiRIT and HiRIT, respectively) on changes in bone mineral density (BMD) in postmenopausal women with osteoporosis.

**Methods:**

Randomized controlled trials that compared the intervention effects of MiRIT and HiRIT were used as selection criteria to assess study patients with osteoporosis or an osteoporotic condition. Database searches were conducted on August 25, 2022, using CENTRAL, PubMed, CINAHL Web of Science, EMBASE, and MEDLINE. A risk of bias assessment was performed using Revised Cochrane risk of bias tool for the assessment of randomized controlled trials. Point estimates and 95 % confidence intervals of change in BMD derived using dual-energy X-ray absorptiometry were collected as outcomes, and a meta-analysis was performed using the amount of change in BMD before and after the intervention. Adverse event data were also collected.

**Results:**

The search yielded six studies (391 patients, mean age 53–65 years) that met the inclusion criteria. The intervention duration ranged from 24 weeks to 13 months. Compared with the MiRIT group, the HiRIT group showed significantly improved BMD of the lumbar spine (standardized mean difference 2.37 [0.10–4.65]). However, a high degree of heterogeneity was observed for three studies (154 patients, I^2^ = 98 %). Almost all studies reported minimal adverse events. The certainty of evidence was extremely low because of the risk of bias, inconsistency among studies, and imprecision in terms of sample size.

**Conclusion:**

Postmenopausal women with osteoporosis may achieve more significantly improved lumbar spine BMD with HiRIT than with MiRIT.

## Highlights

• The effects of moderate versus high-intensity training on bone mineral density in postmenopausal woman were compared.
• High-intensity training would be more beneficial for improving bone mineral density of the lumbar spine than moderate one.
• Almost all studies reported minimal adverse events.
• However, the lack of certainty associated with the evidence should be considered for clinical application.

## 1 Introduction

As life expectancy and the aging population continue to increase globally, age-related diseases are becoming a growing concern (Vos et al., 2017). Osteoporosis is associated with several factors, including aging and menopause (Khosla and Hofbauer, 2017). It is one of the most common chronic metabolic bone diseases and is characterized by decreased bone mineral density (BMD) and increased bone fragility (Sözen et al., 2017). In context of the postmenopausal decrease in estrogen levels, osteoporosis is highly prevalent in women (Tella and Gallagher, 2014), with a global prevalence rate of 23.1 % among women, according to a recent meta-analysis (Salari et al., 2021). Osteoporosis reduces BMD, thereby increasing the risk of fracture resulting from a fall (Johnell et al., 2005; Stone et al., 2003). Dual-energy X-ray absorptiometry is one of the most common methods for measuring areal BMD (aBMD) (Sözen et al., 2017). Furthermore, preventing aBMD decline is essential for controlling the surge in medical costs caused by osteoporosis-related falls and fractures as well as silent fractures, such as vertebral compression fractures (Hernlund et al., 2013).

Exercise therapy is an important management strategy for maintaining BMD (Sözen et al., 2017; Tella and Gallagher, 2014). Several systematic reviews have summarized intervention studies focused on the effects of exercise therapy on BMD, primarily in postmenopausal women (Howe et al., 2011; Anupama et al., 2020; Zitzmann et al., 2022). However, their findings were inconsistent, most likely due to how the activity mode was classified, in addition to intensity, participant inclusion criteria, and other variables. In summary, more frequent exercise is beneficial for improving BMD, but the potential benefits of increased exercise intensity for improving BMD remain unclear. Recently, the efficacy and safety of moderate- to high-intensity exercise therapy have been studied more than the traditional low-intensity exercise therapy that prioritizes safety (Kistler-Fischbacher et al., 2021a; Kistler-Fischbacher et al., 2021b). A review of these studies showed that high-intensity exercise therapy was more effective for improving BMD of the lumbar spine (LS) than moderate- or low-intensity exercise therapy (mean difference 0.031 g/cm^2^, 0.012 g/cm^2^, and 0.010 g/cm^2^, respectively) (Kistler-Fischbacher et al., 2021b). Conversely, there was no effect of high-intensity exercise therapy on the femoral neck (FN) (Kistler-Fischbacher et al., 2021b).

In the past, high-intensity resistance and impact training (HiRIT) was avoided and rarely reported owing to concerns that certain adverse events, such as exercise-related musculoskeletal symptoms, would occur (Uusi-Rasi et al., 2003). However, one study investigated the efficacy and safety of moderate-intensity exercise therapy over the past two decades (Kistler-Fischbacher et al., 2021b), and recently, an increasing number of randomized controlled trials (RCTs) have focused on the efficacy of HiRIT (Hettchen et al., 2021; Harding et al., 2020; Kistler-Fischbacher et al., 2021c). Although the effect of HiRIT on BMD has been extensively reviewed systematically, several concerns remain regarding the lack of reproducibility of screening (Kistler-Fischbacher et al., 2021a; Kistler-Fischbacher et al., 2021b) and the ambiguity of criteria for exercise prescription in the control group (Kitsuda et al., 2021). When conducting a systematic review, it is generally highly recommended to present a complete search strategy and to predefine the details of the control group (Page et al., 2021; McKenzie et al., 2022). Moreover, because these meta-analyses did not include results from relatively new RCTs (Kistler-Fischbacher et al., 2021c; Hettchen et al., 2021), a new meta-analysis that considers all such concerns may provide different results.

This systematic review aimed to examine the effects of moderate-intensity resistance and impact training (MiRIT) and HiRIT on aBMD, particularly in postmenopausal women with low aBMD who are likely to experience a decline in activities of daily living and quality of life with the progression of osteoporosis.

## 2 Materials and methods

### 2.1 Inclusion/exclusion criteria and definitions

This systematic review considered RCTs (published in English) that compared postmenopausal women treated with some form of HiRIT to those treated with MiRIT. The inclusion criteria were as follows: intervention studies focusing on postmenopausal women; study participants with an osteoporotic condition or osteoporosis (an aBMD T-score of <1 standard deviation or <80 % of the young adult mean); groups of study participants either treated or not treated with drug therapy for osteoporosis; MiRIT or HiRIT prescription in any study intervention group; and study outcomes that included aBMD of the LS and FN. Dual-energy X-ray absorptiometry was used as the assessment method for aBMD for all groups. Both vertebral and femoral aBMD are considered useful predictors of fracture and represent clinically relevant outcomes (Marshall et al., 1996). The exclusion criteria were as follows: any diseases affecting bone metabolism (HIV infection, cancer, Gaucher disease, etc.); uncontrolled cardiovascular disease; cognitive disorder based on a Mini-Mental State Examination score of <24; treatment within 3 months after lower extremity surgery or injury; and treatment immediately after lower back injury or onset of localized pain.

The intensities of resistance and impact training were defined as follows: HiRIT was a training load of 80 % of 1 repetition maximum (RM) for ≤6 repetitions; ground reaction force ≥4 times body weight; body weight jumps with stiff-legged landing; and aerobics performed with a load > 4 times body weight. MiRIT was considered the control intervention in this systematic review and defined as follows: a training load of 60 %–80 % of 1 RM 8–15 times; ground reaction force > 2 times to <4 times body weight; heel drop exercise (dorsiflexion ≥ 0°, heel lift); and aerobics performed with a load < 4 times body weight (Kistler-Fischbacher et al., 2021a). If multiple exercise interventions were combined in each group, the exercise with the highest intensity was considered for inclusion in this review. Data on the change in aBMD of the LS or FN before and after the exercise intervention period were collected. Adverse event data were also collected.

### 2.2 Database search methods

To identify exercise trials, the following databases were searched on April 23, 2021, and updated on August 25, 2022: Cochrane Central Register of Controlled Trials (CENTRAL, via The Cochrane Library); PubMed; Cumulative Index to Nursing and Allied Health Literature (CINAHL); Web of Science; EMBASE (via ProQuest Dialog); and MEDLINE (via ProQuest Dialog). Search keywords were “menopause”; “female”; “exercise”; “sports”; “randomized controlled trial”; and “physical fitness.” Appendices 1–6 show the search strategies. This study was registered in the https://www.protocols.io/ domain at https://www.protocols.io/view/the-effectiveness-of-high-intensity-exercise-thera-6qpvrd7rpgmk/v1. In addition, we searched the citation lists of included studies and trial registries and then contacted the authors of the included studies who did not provide sufficient data for our review to obtain additional studies and data if needed.

### 2.3 Data collection

For the study selection, at least two authors (KH and TD) independently reviewed the eligibility criteria for abstracts for inclusion by following an a priori registered protocol.

The titles and abstracts of all potentially relevant studies generated by the search were screened based on the types of study, participants, interventions, and outcome measurements. The full-text articles selected by title and abstract screening were assessed for eligibility. Any disagreements were resolved through consensus by the two authors (KH and TD) or by the third author (TK).

Two authors (KH and TD) independently extracted data using a customized data extraction form for data extraction and management. The form contained information on participant characteristics, including initial sample size, dropout rate, mean participant age, mean body mass index, BMD outcomes, medication-related information, and adverse events. Another form contained information about exercise characteristics, including the number of recruited/analyzed participants, duration (supervision) of exercise, types of exercise, frequency of exercise, intensity of exercise, other details of exercise in the intervention group, and detailed exercise information in the control group. The two authors (KH and TD) also extracted the details required to assess the risk of bias. Again, any disagreements were discussed by the two authors to reach a consensus, with the arbiter (TK), if required. The authors of studies in which data were inadequately reported were contacted for further clarification.

### 2.4 Assessment of risk of bias and quality of evidence

To assess the risk of bias in the included studies, two reviewers (KH and TK) independently evaluated the risk of bias using Revised Cochrane risk of bias tool for randomized trials (Sterne et al., 2019). Each study was reviewed according to the following domains: (1) bias resulting from the randomization process; (2) bias caused by deviations from intended interventions; (3) bias caused by missing outcome data; (4) bias in outcome measurement; and (5) bias in selecting the reported result. Each study was evaluated as low risk of bias, some concerns of bias, or a high risk of bias. Two authors (KH and TK) discussed any disagreements with a third author (SY) as arbiter, if necessary.

A table summarizing the findings for aBMD was generated (Table 1). Grading was used to evaluate the quality of evidence using the Grading of Recommendations Assessment, Development, and Evaluation approach for the summary of findings (Table 1) (G. Guyatt et al., 2011). The quality of evidence was determined by one author (TK) and then confirmed and finalized by another author (SY).

**Table 1:** Summary of findings for HiRIT versus MiRIT in postmenopausal women with osteoporosis.

Study population: Postmenopausal women with osteoporosis or an osteoporotic condition Setting: Community dwelling Intervention: High-intensity resistance and impact training Control: Moderate-intensity resistance and impact training					
Outcomes	Anticipated absolute effects⁎ (95 % CI)		No of participants (studies)	Certainty of evidence (GRADE)	Comments
	MiRIT	HiRIT			
aBMD change: lumbar spine assessed with DEXA scan	SMD ranged across the control group from −1.2 to 0 SD	SMD 2.37 SD higher (0.1 higher to 4.65 higher)	240 (3 RCTs)	⨁◯◯◯ Very low^a,b,c^	HiRIT increases the bone mineral density of the lumbar spine
aBMD change: femoral neck assessed with DEXA scan	SMD ranged across the control group from −2 to −0.006 SD	SMD 1.38 SD higher(0.08 lower to 2.85 higher)	242 (3 RCTs)	⨁◯◯◯ Very low^a,b,c^	HiRIT likely increases bone mineral density of the femoral neck
aBMD: areal bone mineral density; DEXA: dual-energy X-ray absorptiometry; GRADE: Grading of Recommendations, Assessment, Development and Evaluations; HiRIT: high-intensity resistance and impact training; MiRIT: moderate-intensity resistance and impact training; RCTs: randomized controlled trials; SD: standard deviation; SMD: standardized mean difference					
Grades of evidence as defined by GRADE Working Group High certainty: We are very confident that the true effect lies close to that of the estimate of the effect. Moderate certainty: We are moderately confident in the effect estimate: the true effect is likely to be close to the estimate of the effect, but there is a possibility that it is substantially different. Low certainty: Our confidence in the effect estimate is limited: the true effect may be substantially different from the estimate of the effect. Very low certainty: We have very little confidence in the effect estimate: the true effect is likely to be substantially different from the estimate of the effect.					

⁎ Each outcome was downgraded for (a) serious risk of bias, (b) serious inconsistency, and (c) serious imprecision.

### 2.5 Data analysis

For measures of the treatment effect, we pooled the mean differences and 95 % confidence intervals (CIs) for the continuous variables (reporting mean and standard deviation or standard error of the mean) for each trial. We also summarized the adverse event data.

Meta-analyses were conducted on those outcomes for which the amount of change in the outcome before and after the intervention could be extracted. For cases in which the units of outcome were different, we attempted to integrate the data by calculating the standardized mean difference (SMD). For cases in which substantial heterogeneity was present (I^2^ > 50 %), we assessed the reason for such heterogeneity. The Cochrane chi-squared test (Q test) was used for the I^2^ statistic, and a p value of <0.10 was considered statistically significant (Higgins et al., 2022).

To assess reporting biases, we intended to assess the possibility of publication bias using funnel plots if there were >10 studies (Sterne et al., 2011). Meta-analysis was performed using Review Manager software (RevMan 5.4, Cochrane). Because the size of intervention effects varied owing to differences in settings across studies, we decided to use a random-effects model. If the mean difference and 95 % CI were not reported, the study was excluded from the meta-analysis.

To explain the influence that effect modifiers can have on results, we conducted subgroup analyses of the aBMD. On collecting sufficient data, we planned to divide the study participants into two groups based on an age of either <60 years or ≥60 years to examine the difference in effect by age. To confirm the robustness of the results, we conducted a meta-analysis of studies by omitting the high risk of bias and changing the model from a random-effects model to a fixed-effects model in sensitivity analysis.

## 3 Results

### 3.1 Description of studies

**Fig. 1:**
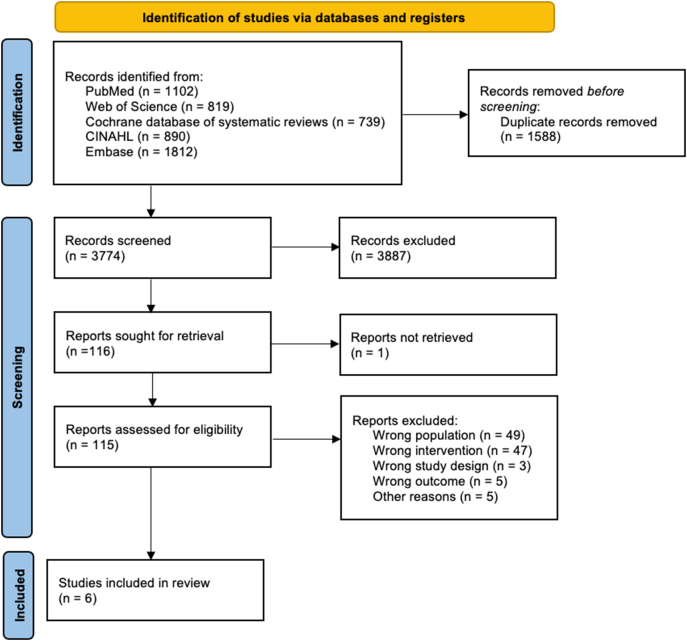
Overview of the study selection process.

The initial search result identified 3774 studies, which were screened for eligibility after removing duplicates. A total of 115 full-text articles were screened for eligibility based on their title and abstract, of which 109 were excluded and 6 were included (Hettchen et al., 2021; Kistler-Fischbacher et al., 2021c; Brentano et al., 2008; Murtezani et al., 2014; Watson et al., 2018; Sen et al., 2020). Fig. 1 illustrates an overview of the study selection process.

Table 2 outlines the participant characteristics for each of the included studies. The initial sample size per group varied between 19 (Brentano et al., 2008) and 115 (Kistler-Fischbacher et al., 2021c) participants. The highest dropout rate was 24.1 % (Hettchen et al., 2021). The average age of participants was 60 years (mean age 53.1–65.0 years). All six studies measured the aBMD of the LS. Some studies used pharmacotherapy. To ensure that no differences in the effects of exercise therapy between the HiRIT and MiRIT groups would arise during the randomization process, the authors of the six studies used stratified randomization. Almost all studies reported minimal adverse events, such as mild muscle strain or falls (Hettchen et al., 2021; Kistler-Fischbacher et al., 2021c; Murtezani et al., 2014; Watson et al., 2018; Sen et al., 2020). A meta-analysis of adverse events could not be performed because the types of outcomes and measurement methods varied widely among the studies. Of the six studies, four reported adverse events, including falls, injury, fracture, and pain. The studies were conducted in Brazil (Brentano et al., 2008), Serbia (Murtezani et al., 2014), Australia (Kistler-Fischbacher et al., 2021c; Watson et al., 2018), Turkey (Sen et al., 2020), and Germany (Hettchen et al., 2021).

**Table 2:** Participant characteristics across the six randomized controlled trials.

Authors (year)	Initial sample size (n)	Dropout (%)	Mean age of participants (years)	Mean body mass index of participants	Bone mineral density outcomes	Medication	Adverse events
Brentano et al. (2008)	19	0	Not mentioned	Not mentioned	LS, trochanter of femur, intertrochanter, FN, and Ward's triangle	14 participants were taking hormone therapy. (Each group assumed hormone therapy as a covariant.)	Not mentioned
Murtezani et al. (2014)	64	4.7	HiRIT: 59.778 ± 5.996 MiRIT: 60.68 ± 7.62	HiRIT: 41.41 ± 3.58 MiRIT: 41.104 ± 3.46	LS	Not mentioned	HiRIT group: 1 participant withdrew spontaneously because of injury post-randomization
Watson et al. (2018)	101	14.85	HiRIT: 65 ± 5 MiRIT: 65 ± 5	HiRIT: 23.7 ± 3.2 MiRIT: 24.5 ± 4.6	LS and FN	HiRIT and MiRIT groups: 10 participants in each group took any of the following medications—bisphosphonate, denosumab, and hormone therapy. (Stratified randomization was conducted based on osteoporosis medication.)	HiRIT group: 1 participant experienced a mild low-back muscle strain HiRIT and MiRIT groups: 7 participants (5 HiRIT; 2 MiRIT) fell during the trial; none of these falls resulted in injury to the participant; all falls occurred outside of the exercise sessions.
Sen et al. (2020)	38	18.4	HiRIT: 53.1 ± 4.4 MiRIT: 55.0 ± 4.6	HiRIT: 26.5 ± 3.9 MiRIT: 26.6 ± 2.7	LS, FN, and total hip	Not mentioned	No severe adverse events, such as fall-related fractures or new onset local spine pain, were observed during the study period.
Hettchen et al. (2021)	54	24.1	HiRIT: 53.6 ± 2.0 MiRIT: 54.5 ± 1.6	HiRIT: 23.7 ± 3.4 MiRIT: 24.9 ± 4.8	LS and total hip	No between-group differences in pharmacologic therapy that could have affected results were observed.	There were no changes in disease affecting this study outcome or absences >2 weeks from exercise.
Kistler-Fischbacher et al., 2021c	115	9.6	HiRIT: 63.3 ± 6.4 MiRIT: 63.6 ± 4.9	HiRIT: 26.2 ± 4.6 MiRIT: 25.5 ± 4.5	LS; FN; trochanter of femur; total hip; ultra-distal; one-third, mid, and total radius; and whole body	Participants taking bisphosphonates (alendronate, risedronate, and zoledronic acid) or denosumab for ≥12 months before enrollment were targeted for recruitment. (A block randomization procedure was used to assign groups based on whether bone medications were taken at baseline.)	HiRIT group: 15 participants (14 fell once, 1 fell twice) MiRIT group: 12 participants (10 fell once, 2 fell twice) fell during the trial. HiRIT and MiRIT groups: 1 participant in each group sustained fractures during the intervention.

LS: lumbar spine; FN: femoral neck; HiRIT: high-intensity resistance and impact training; MiRIT: moderate-intensity resistance and impact training.

### 3.2 Exercise interventions

The exercise intervention lasted from 24 weeks (Brentano et al., 2008; Sen et al., 2020) to 13 months (Hettchen et al., 2021). HiRIT consisted of resistance and muscle-strengthening exercises as well as jumping movements. The frequency of exercise in all included studies was twice or thrice weekly. Before a high-intensity exercise, warm-up exercise was generally performed to prevent injuries and other adverse events. In the intervention group, exercise therapy primarily consisted of high-intensity muscle-strengthening exercises and vertical jumping, whereas in the control group, exercise therapy included not only moderate-intensity muscle-strengthening exercises but also aquatic exercise (Murtezani et al., 2014) and exercise with vibrations (Sen et al., 2020) (Table 3).

**Table 3:** Characteristics of the exercises included in the six randomized controlled trials.

Authors (year)	Number of recruited/analyzed participants	(Supervision) duration	Types of exercise	Frequency of exercise	Intensity of exercise	Other exercise details for the intervention group	Exercise details for the control group		
Brentano et al. (2008)	HiRIT: 10/10 MiRIT: 9/9	24 weeks	Muscle strength exercises: leg press, hip abduction, hip adduction, knee extension, chest fly, reverse fly, arm curl, triceps push-down, sit-ups, and back extension	HiRIT: 3 sessions/week MiRIT: 3 sessions/week	Each exercise was performed 2–4 times with 6–20 repetitions and 45 %–80 % of 1 RM	1 RM test performed every 8 weeks to verify muscular strength changes	2–3 sets and 20–10 repetitions with 45 %–60 % of 1 RM		
Murtezani et al. (2014)	HiRIT: 33/31 MiRIT: 31/30	10 months	Core exercise set: aerobic weight-bearing exercises and progressive, resistive exercises	HiRIT: 3 sessions/week MiRIT: 3 sessions/week	In the prone position, a backpack containing weights equivalent to 30 % of the maximum strength of the back extensors was used (2 sets of 6–8 repetitions at 70 % or 80 % of 1 RM)	Each session: 10-min warm-up, 35-min exercise training, and 10-min cool down	10-min warm-up, 15-min weight-resistance training (aquatic exercise), and 10-min cool down		
Watson et al. (2018)	HiRIT: 49/43 MiRIT: 52/43	8 months	Exercises in the first intervention month: body weight and low intensity Exercises in the subsequent months: resistance exercises (deadlift, overhead press, and back squat) performed at high intensity	HiRIT: 2 sessions/week MiRIT: 3 sessions/week	5 sets of 5 repetitions, maintaining an intensity of 80 %–85 % of 1 RM	30-min exercise with supervision	30-min, 2 sessions/week program of moderate-intensity workouts (10–15 repetitions at 60 % of 1 RM) performed at home for 8 months Exercise program improved balance and mobility but provided minimal bone stimulation		
Sen et al. (2020)	HiRIT: 19/16 MiRIT: 19/15	24 weeks	After the initial training program, vertical jumps with a jump rope on 2 legs were performed	HiRIT: 3 sessions/week MiRIT: 3 sessions/week	From a minimum of 10 jumps/session to a maximum of 60 jumps/set over 12 weeks, the total number of jumps was increased by 5 jumps/week	Initial training program: warm-up (cycling and stepping), stretching, and exercises for hip, knee, and back extensor	After the initial training program, participants received vibrations under supervision while squatting, deep squatting, wide-step squatting, lunging, and hands-front lunging		
Hettchen et al. (2021)	HiRIT: 27/21 MiRIT: 27/20	13 months	Weight-bearing exercise: aerobic dancing, jump training, and resistance training	HiRIT: 3 sessions/week MiRIT: not mentioned	In 1 session/week, high-intensity phases (80 %–85 % of HR_max_) were interspersed with 60 s of lower intensity (65 %–70 %) exercise Second session employed a corresponding 30-s/30-s protocol	First 4 weeks of exercise: briefing, familiarization, correct movements and lifting techniques, body sensation, and using rate-of-perceived-exertion approach	Program of 2 rounds of 12 weeks of supervised group exercises, followed by 12–14 weeks of non-supervised, video-guided home exercises Supervised group exercise session began with walking or marching for 15 min, followed by stretching and floor exercises for 20 min, and 10-min cool down		
Kistler-Fischbacher et al., 2021c	HiRIT: 57/52 MiRIT: 58/52	8 months	Modules of three free-weight-resistance training exercises (deadlift, back squat, and overhead press), one high-impact exercise (jump drop), and two balance exercises that were changed per session	HiRIT: 2 sessions/week MiRIT: 2 sessions/week	A total of 5 sets of 5 repetitions were performed after a familiarization period Resistance training load was increased continuously to achieve and maintain a training intensity of 80 %–85 % of 1 RM or a rating of ≥16 (i.e., “very hard”) on a 6- to 20-point Borg scale of perceived exertion	Jump drop loading began with 2 weeks of heel drops and then progressed to a jump drop with stiff-legged landing and fully extended knees and hips. After 2 weeks, jump and drop landing heights were increased, and participants were instructed to use their arms to pull themselves up to a pull-up bar, to assist and control the jump	Exercises and movement principles from Pilates and functional movement combined with balance and therapeutic exercises to improve whole-body strength, balance, mobility, and posture throughout the program		

RM: repetition maximum; HR_max_: heart rate maximum; HiRIT: high-intensity resistance and impact training; MiRIT: moderate-intensity resistance and impact training.

### 3.3 Risk of bias

**Fig. 2:**
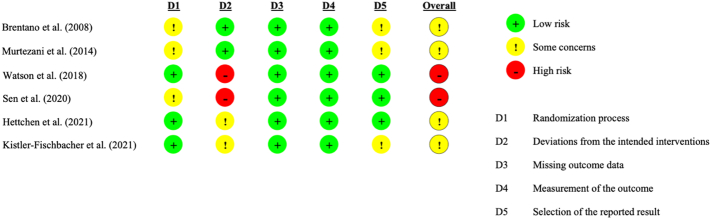
Risk of bias assessment for each study according to the domain.

Fig. 2 shows the risk of bias assessment for each study according to the domain.

Of the six studies reviewed, two had a high risk of bias owing to the risk of deviations from the intended interventions. The remaining four studies were believed to have some concerns for the risk of bias because of inadequacies in the randomization process, deviations from the intended interventions, and selection of the reported results.

### 3.4 Effects of HiRIT

**Fig. 3:**
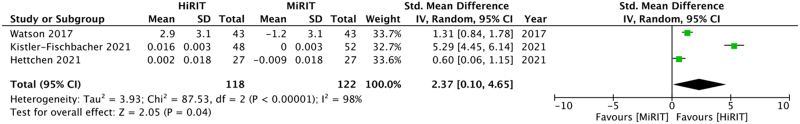
Forest plots of meta-analyses of the effects of an exercise intervention on the bone mineral density of the lumbar spine.

Of the six RCTs that met the inclusion criteria, aBMD of the LS and FN was reported in three RCTs with 118 participants (Hettchen et al., 2021; Kistler-Fischbacher et al., 2021c; Watson et al., 2018). Of these three studies, two reported aBMD as absolute densities (Hettchen et al., 2021; Kistler-Fischbacher et al., 2021c), whereas one reported aBMD as a percentage change from baseline aBMD (Watson et al., 2018). Because of these differences in the units of measurement, SMD was calculated during the meta-analysis. Compared with the control group, the HiRIT group showed significantly improved aBMD of the LS (SMD 2.37; 95 % CI, 0.10–4.65); however, heterogeneity was high (Fig. 3).

**Fig. 4:**
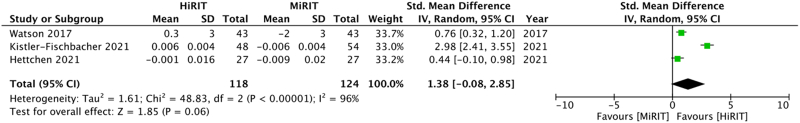
Forest plots of meta-analyses of the effects of an exercise intervention on the bone mineral density of the femoral neck.

Results of the intervention and control groups are shown as a pooled SMD with a 95 % CI. Conversely, aBMD of the FN did not differ significantly between the HiRIT and MiRIT groups (SMD 1.38; 95 % CI, −0.08–2.85) (Fig. 4).

Results of the intervention and control groups are shown as a pooled SMD with a 95 % CI.

In a subgroup analysis of two studies in which the mean participant age was ≥60 years (Kistler-Fischbacher et al., 2021c; Watson et al., 2018), the change in aBMD of the LS did not differ significantly between the HiRIT and MiRIT groups. Similarly, the change in aBMD of the FN showed no significant differences between the two groups. For participants with a mean age of <60 years, a subgroup meta-analysis could not be performed because only one study was found with this age group. A sensitivity analysis comparing aBMD changes in the two studies (Hettchen et al., 2021; Kistler-Fischbacher et al., 2021c), excluding those with a high risk of bias, showed that the effect of LS/FN on aBMD was not significantly different between the HiRIT and MiRIT groups. According to the results of a fixed-effects model analysis of differences in the effect sizes of the interventions in the three studies, HiRIT was found to be beneficial for the aBMD of both LS and FN.

### 3.5 Grading of evidence

Table 1 summarizes the certainty of evidence. Of the three studies included in the meta-analysis, one had a high risk of bias, whereas two had some concerns for risk of bias, which thus downgraded the certainty of evidence. As shown in Fig. 3, Fig. 4, statistical heterogeneity was observed in the integrated results of the two meta-analyses, which were found to be inconsistent, and the certainty of evidence was downgraded based on this finding. Further downgrading because of imprecision was also considered based on the small total sample size. Finally, the certainty of evidence of the efficacy of HiRIT compared with that of MiRIT for aBMD was found to be very low. Because fewer than 10 studies were included in aBMD comparisons, a funnel plot was not graphed.

## 4 Discussion

### 4.1 Summary of main results

Based on data from six RCTs involving 391 participants, we compared the efficacy of HiRIT with that of MiRIT for aBMD in postmenopausal women with osteoporosis. We also examined the effect of age separately. Our results showed that HiRIT tended to be more effective than MiRIT; however, the certainty of evidence was low, primarily because of a high degree of heterogeneity. Comparison of the effects of HiRIT and MiRIT on aBMD showed that HiRIT was significantly more effective than MiRIT for LS (SMD 2.37; 95 % CI, 0.10–4.65), but their effects did not significantly differ for FN (SMD 1.38; 95 % CI, −0.08–2.85). This inference was also weakened because these integrated results were accompanied by heterogeneity, as evidenced by the large change in 95 % CI in the sensitivity analysis, the slightly different ages of the participants across the studies, and the differences in intervention details. Subgroup analyses based on participant age allowed us to perform a meta-analysis with two studies that were restricted to older participants (Kistler-Fischbacher et al., 2021c; Watson et al., 2018). No significant differences were observed between groups based on the subgroup analyses, and a high degree of statistical heterogeneity remained. Because the two studies were also relatively similar in terms of baseline participant characteristics and the exercise prescription for the intervention group, the unexplained heterogeneity remained.

### 4.2 Overall completeness and applicability of evidence

One review recommended a regular weight-bearing exercise regimen (Sözen et al., 2017), and recent guidelines from the United Kingdom targeting individuals aged >50 years (Compston et al., 2017) recommended weight-bearing exercise, regular weight-bearing exercise, muscle strengthening, and balance training exercise interventions, among other approaches. However, the specific load intensity was not clearly defined. To integrate data on efficacy, our review focused on HiRIT, which has been increasingly reported in recent years. The results of the primary meta-analysis suggest that HiRIT is more effective than MiRIT for improving the aBMD of LS. However, the interpretation of these results and application of HiRIT require caution because concerns remain regarding heterogeneity, insufficient robustness of the results, and low certainty of evidence. We did not find statistically significant differences for FN aBMD; therefore, it is unlikely that HiRIT is more effective than MiRIT for improving the aBMD of FN. In addition, the final sample sizes included for each outcome were also insufficient, resulting in low certainty of evidence (G.H. Guyatt et al., 2011). Further RCTs are warranted to address these issues. Finally, based on the results of this review, it is impossible to make specific recommendations regarding the optimal intensity of exercise therapy.

### 4.3 Quality of the evidence

There were some concerns about the overall quality of the included studies; therefore, the results of this review must be interpreted with caution. Particularly in the domain of deviations from the intended interventions, two studies were found to have high risk of bias (Watson et al., 2018; Sen et al., 2020). In some cases, blinding the patients and care providers to the intervention type may be difficult in exercise therapy. However, this bias may not affect aBMD outcomes. Some concerns in the randomization process were found in three studies (Brentano et al., 2008; Murtezani et al., 2014; Sen et al., 2020). In general, randomization is a task that should be approached with caution. This should be addressed in future RCTs. Some concerns about the selection of the reported results were also found in three studies (Kistler-Fischbacher et al., 2021c; Brentano et al., 2008; Murtezani et al., 2014). The *spin problem*—hiding potentially negative results or selectively “cherry-picking” specific results—has been recently reported (Boutron and Ravaud, 2018). In conducting new RCTs related to this review question, researchers should develop protocols in advance and avoid selective outcome reporting biases, such as “p value hacking” (Head et al., 2015).

### 4.4 Agreements and disagreements with other studies or reviews

Exercise therapy is beneficial for BMD in postmenopausal women with osteoporosis (Sözen et al., 2017; Howe et al., 2011; Anupama et al., 2020; Compston et al., 2017). High-intensity exercise is more effective than low-intensity exercise, regardless of bone density status (Kistler-Fischbacher et al., 2021a). In addition, results from a meta-analysis demonstrated that the effect was large (Chittaranjan Andrade, 2015). A meta-analysis that examined the effect of high-intensity exercise therapy on BMD in LS and FN found that this type of exercise was effective for LS but not for FN, which supported our findings (Kistler-Fischbacher et al., 2021b). In women, BMD generally peaks around the age of 18 years, after which it gradually declines (Orito et al., 2009). The rate of BMD decrease is particularly significant after menopause. Moreover, a study found that bone loss nearly tripled during the first 10 years after menopause (Warming et al., 2002). Future reviews with finer stratification of postmenopausal age may yield new findings. In this review, we included two RCTs that were not considered in previous meta-analyses (Hettchen et al., 2021; Kistler-Fischbacher et al., 2021c), and the results were similar to those of previous reviews, even if focused on women with reduced BMD. According to the findings of a meta-analysis that examined the effect of exercising more than once a week on individuals taking pharmacological agents to treat osteoporosis, exercising more than twice a week is more beneficial for improving aBMD than exercising once a week (Zitzmann et al., 2022). In the studies included in this review, exercise therapy was implemented twice or thrice weekly, and this level of frequency was considered desirable to prevent dropout/overwork resulting from excessive frequency of exercise (Hettchen et al., 2021; Kistler-Fischbacher et al., 2021c; Brentano et al., 2008; Murtezani et al., 2014; Watson et al., 2018; Sen et al., 2020). Based on the findings of the present review, performing HiRIT more than twice weekly is relatively effective for improving the aBMD of the LS, considering that the possible adverse events are few.

### 4.5 Limitations

This systematic review has several limitations. First, although the frequency of interventions was generally similar across studies, the duration of interventions varied greatly, ranging 6–13 months. The lack of a clear prior restriction on the duration of interventions may have contributed to the heterogeneity of results. We used the BMD at the final follow-up of each trial for data integration because the follow-up period was not specified in our protocol. However, specific follow-up time points should have been specified in advance. Second, >50 % of the studies included in this review were conducted in Europe. Further international validation is required to make the findings more generalizable to a wider range of countries and regions. Third, we collected data from articles published only in the English language because of the limited resources for this study; thus, other evidence may have been excluded. Moreover, because only a few studies were included, we did not use predefined statistical tests to determine whether publication bias existed. Given that we did not search unpublished studies or gray literature, publication bias is likely. A more comprehensive search strategy should be developed for future reviews. Nevertheless, another meta-analysis similarly demonstrated the efficacy of high-load resistance training, mainly in BMD of the LS, similar to our results (Kitsuda et al., 2021).

## 5 Conclusions

In conclusion, our findings indicate that HiRIT is more effective for improving the aBMD of LS than MiRIT in postmenopausal women with osteoporosis. Future research should collect data from RCTs with a sufficiently large sample size to allow for an analysis of specific participant types and a more standardized HiRIT intervention.

## CRediT authorship contribution statement

This review was conceived by all the authors. TK and SY searched for articles. KH and TD screened articles. KH and TD extracted data and TK checked them. KH and TK assessed the risk of bias and SY checked them. TK analyzed the data. KH and TK wrote the first draft of the manuscript. All authors contributed to the interpretation of results, manuscript preparation, and revisions. All authors have read and agreed to the published version of the manuscript.

## Declaration of competing interest

The authors declare no conflict of interest.

## Data Availability

Data will be made available on request.

## Appendix A Supplementary data

Literature search strategies Image 1

## References

[bb0005] Anupama D.S., Norohna J.A., Acharya K.K., Ravishankar G., George A. (2020). Effect of exercise on bone mineral density and quality of life among postmenopausal women with osteoporosis without fracture: a systematic review. Int. J. Orthop. Trauma Nurs..

[bb0010] Boutron I., Ravaud P. (2018). Misrepresentation and distortion of research in biomedical literature. Proc. Natl. Acad. Sci. U. S. A..

[bb0015] Brentano M.A., Cadore E.L., Da Silva E.M., Ambrosini A.B., Coertjens M., Petkowicz R., Viero I., Kruel L.F.M. (2008). Physiological adaptations to strength and circuit training in postmenopausal women with bone loss. J. Strength Cond. Res..

[bb0020] Chittaranjan Andrade M. (2015). Mean difference, standardized mean difference (SMD), and their use in meta-analysis: as simple as it gets. J. Clin. Psychiatry.

[bb0025] Compston J., Cooper A., Cooper C., Gittoes N., Gregson C., Harvey N., Hope S., Kanis J.A., McCloskey E.V., Poole K.E.S., Reid D.M., Selby P., Thompson F., Thurston A., Vine N., National Osteoporosis Guideline Group (NOGG) (2017). UK clinical guideline for the prevention and treatment of osteoporosis. Arch. Osteoporos..

[bb0030] Guyatt G., Oxman A.D., Akl E.A., Kunz R., Vist G., Brozek J., Norris S., Falck-Ytter Y., Glasziou P., Debeer H., Jaeschke R., Rind D., Meerpohl J., Dahm P., Schünemann H.J. (2011). GRADE guidelines, 1. Introduction – GRADE evidence profiles and summary of findings tables. J. Clin. Epidemiol..

[bb0035] Guyatt G.H., Oxman A.D., Kunz R., Brozek J., Alonso-Coello P., Rind D., Devereaux P.J., Montori V.M., Freyschuss B., Vist G., Jaeschke R., Williams J.W., Murad M.H., Sinclair D., Falck-Ytter Y., Meerpohl J., Whittington C., Thorlund K., Andrews J., Schünemann H.J. (2011). GRADE guidelines 6. Rating the quality of evidence – imprecision. J. Clin. Epidemiol..

[bb0040] Harding A.T., Weeks B.K., Lambert C., Watson S.L., Weis L.J., Beck B.R. (2020). A comparison of bone-targeted exercise strategies to reduce fracture risk in middle-aged and older men with osteopenia and osteoporosis: LIFTMOR-M semi-randomized controlled trial. J. Bone Miner. Res..

[bb0045] Head M.L., Holman L., Lanfear R., Kahn A.T., Jennions M.D. (2015). The extent and consequences of P-hacking in science. PLoS Biol..

[bb0050] Hernlund E., Svedbom A., Ivergård M., Compston J., Cooper C., Stenmark J., McCloskey E.V., Jönsson B., Kanis J.A. (2013). Osteoporosis in the European Union: medical management, epidemiology and economic burden: a report prepared in collaboration with the international osteoporosis foundation (IOF) and the European Federation of Pharmaceutical Industry Associations (EFPIA). Arch. Osteoporos..

[bb0055] Hettchen M., von Stengel S., Kohl M., Murphy M.H., Shojaa M., Ghasemikaram M., Bragonzoni L., Benvenuti F., Ripamonti C., Benedetti M.G., Julin M., Risto T., Kemmler W. (2021). Changes in menopausal risk factors in early postmenopausal osteopenic women after 13 months of high-intensity exercise: the randomized controlled ACTLIFE-RCT. Clin. Interv. Aging.

[bb0060] Higgins J., Thomas J., Chandler J., Cumpston M., Li T., Page M., Welch V. (2022). Cochrane Handbook for Systematic Reviews of Interventions version 6.3.

[bb0065] Howe T.E., Shea B., Dawson L.J., Downie F., Murray A., Ross C., Harbour R.T., Caldwell L.M., Creed G. (2011). Exercise for preventing and treating osteoporosis in postmenopausal women. Cochrane Database Syst. Rev..

[bb0070] Johnell O., Kanis J.A., Oden A., Johansson H., De Laet C., Delmas P., Eisman J.A., Fujiwara S., Kroger H., Mellstrom D., Meunier P.J., Melton L.J., O’Neill T., Pols H., Reeve J., Silman A., Tenenhouse A. (2005). Predictive value of BMD for hip and other fractures. J. Bone Miner. Res..

[bb0075] Khosla S., Hofbauer L.C. (2017). Osteoporosis treatment: recent developments and ongoing challenges. Lancet Diabetes Endocrinol..

[bb0080] Kistler-Fischbacher M., Weeks B.K., Beck B.R. (2021). The effect of exercise intensity on bone in postmenopausal women (part 1): a systematic review. Bone.

[bb0085] Kistler-Fischbacher M., Weeks B.K., Beck B.R. (2021). The effect of exercise intensity on bone in postmenopausal women (part 2): a meta-analysis. Bone.

[bb0090] Kistler-Fischbacher M., Yong J.S., Weeks B.K., Beck B.R. (2021). A comparison of bone-targeted exercise with and without antiresorptive bone medication to reduce indices of fracture risk in postmenopausal women with low bone mass: the MEDEX-OP randomized controlled trial. J. Bone Miner. Res..

[bb0095] Kitsuda Y., Wada T., Noma H., Osaki M., Hagino H. (2021). Impact of high-load resistance training on bone mineral density in osteoporosis and osteopenia: a meta-analysis. J. Bone Miner. Metab..

[bb0100] Marshall D., Johnell O., Wedel H. (1996). Meta-analysis of how well measures of bone mineral density predict occurrence of osteoporotic fractures. Br. Med. J..

[bb0105] McKenzie J., Brennan S., Re R., HJ T., RV J., J T. (2022). Cochrane Handbook for Systematic Reviews of Interventions Version 6.3.

[bb0110] Murtezani A., Nevzati A., Ibraimi Z., Sllamniku S., Meka V.S., Abazi N. (2014). The effect of land versus aquatic exercise program on bone mineral density and physical function in postmenopausal women with osteoporosis: a randomized controlled trial. Ortop. Traumatol. Rehabil..

[bb0115] Orito S., Kuroda T., Onoe Y., Sato Y., Ohta H. (2009). Age-related distribution of bone and skeletal parameters in 1,322 japanese young women. J. Bone Miner. Metab..

[bb0120] Page M.J., Moher D., Bossuyt P.M., Boutron I., Hoffmann T.C., Mulrow C.D., Shamseer L., Tetzlaff J.M., Akl E.A., Brennan S.E., Chou R., Glanville J., Grimshaw J.M., Hróbjartsson A., Lalu M.M., Li T., Loder E.W., Mayo-Wilson E., Mcdonald S., Mcguinness L.A., Stewart L.A., Thomas J., Tricco A.C., Welch V.A., Whiting P., Mckenzie J.E. (2021). PRISMA 2020 explanation and elaboration: updated guidance and exemplars for reporting systematic reviews. BMJ.

[bb0125] Salari N., Ghasemi H., Mohammadi L., Behzadi Mh., Rabieenia E., Shohaimi S., Mohammadi M. (2021). The global prevalence of osteoporosis in the world: a comprehensive systematic review and meta-analysis. J. Orthop. Surg. Res..

[bb0130] Sen E.I., Esmaeilzadeh S., Eskiyurt N. (2020). Effects of whole-body vibration and high impact exercises on the bone metabolism and functional mobility in postmenopausal women. J. Bone Miner. Metab..

[bb0135] Sözen T., Özışık L., Başaran N.Ç. (2017). An overview and management of osteoporosis. Eur. J. Rheumatol..

[bb0140] Sterne J.A.C., Savović J., Page M.J., Elbers R.G., Blencowe N.S., Boutron I., Cates C.J., Cheng H.Y., Corbett M.S., Eldridge S.M., Emberson J.R., Hernán M.A., Hopewell S., Hróbjartsson A., Junqueira D.R., Jüni P., Kirkham J.J., Lasserson T., Li T., McAleenan A., Reeves B.C., Shepperd S., Shrier I., Stewart L.A., Tilling K., White I.R., Whiting P.F., Higgins J.P.T. (2019). RoB 2: a revised tool for assessing risk of bias in randomised trials. BMJ.

[bb0145] Sterne J.A.C., Sutton A.J., Ioannidis J.P.A., Terrin N., Jones D.R., Lau J., Carpenter J., Rücker G., Harbord R.M., Schmid C.H., Tetzlaff J., Deeks J.J., Peters J., Macaskill P., Schwarzer G., Duval S., Altman D.G., Moher D., Higgins J.P.T. (2011). Recommendations for examining and interpreting funnel plot asymmetry in meta-analyses of randomised controlled trials. BMJ.

[bb0150] Stone K.L., Seeley D.G., Lui L.Y., Cauley J.A., Ensrud K., Browner W.S., Nevitt M.C., Cummings S.R., Osteoporotic Fractures Research Group (2003). BMD at multiple sites and risk of fracture of multiple types: Long-term results from the study of osteoporotic fractures. J. Bone Miner. Res..

[bb0155] Tella S.H., Gallagher J.C. (2014). Prevention and treatment of postmenopausal osteoporosis. J. Steroid Biochem. Mol. Biol..

[bb0160] Uusi-Rasi K., Kannus P., Cheng S., Sievänen H., Pasanen M., Heinonen A., Nenonen A., Halleen J., Fuerst T., Genant H., Vuori I. (2003). Effect of alendronate and exercise on bone and physical performance of postmenopausal women: a randomized controlled trial. Bone.

[bb0165] Vos T., Abajobir A.A., Abbafati C., Abbas K.M., Abate K.H., Abd-Allah F., Abdulle A.M., Abebo T.A., Abera S.F., Aboyans V., Abu-Raddad L.J., Ackerman I.N., Adamu A.A., Adetokunboh O., Afarideh M., Afshin A., Agarwal S.K., Aggarwal R., Agrawal A., Agrawal S., Ahmad Kiadaliri A., Ahmadieh H., Ahmed M.B., Aichour A.N., Aichour I., Aichour M.T.E., Aiyar S., Akinyemi R.O., Akseer N., Al Lami F.H., Alahdab F., Al-Aly Z., Alam K., Alam N., Alam T., Alasfoor D., Alene K.A., Ali R., Alizadeh-Navaei R., Alkerwi A., Alla F., Allebeck P., Allen C., Al-Maskari F., Al-Raddadi R., Alsharif U., Alsowaidi S., Altirkawi K.A., Amare A.T., Amini E., Ammar W., Amoako Y.A., Andersen H.H., Antonio C.A.T., Anwari P., Ärnlöv J., Artaman A., Aryal K.K., Asayesh H., Asgedom S.W., Assadi R., Atey T.M., Atnafu N.T., Atre S.R., Avila-Burgos L., Avokpaho E.F.G.A., Awasthi A., Ayala Quintanilla B.P., Ba Saleem H.O., Bacha U., Badawi A., Balakrishnan K., Banerjee A., Bannick M.S., Barac A., Barber R.M., Barker-Collo S.L., Bärnighausen T., Barquera S., Barregard L., Barrero L.H., Basu S., Battista B., Battle K.E., Baune B.T., Bazargan-Hejazi S., Beardsley J., Bedi N., Beghi E., Béjot Y., Bekele B.B., Bell M.L., Bennett D.A., Bensenor I.M., Benson J., Berhane A., Berhe D.F., Bernabé E., Betsu B.D., Beuran M., Beyene A.S., Bhala N., Bhansali A., Bhatt S., Bhutta Z.A., Biadgilign S., Bienhoff K., Bikbov B., Birungi C., Biryukov S., Bisanzio D., Bizuayehu H.M., Boneya D.J., Boufous S., Bourne R.R.A., Brazinova A., Brugha T.S., Buchbinder R., Bulto L.N.B., Bumgarner B.R., Butt Z.A., Cahuana-Hurtado L., Cameron E., Car M., Carabin H., Carapetis J.R., Cárdenas R., Carpenter D.O., Carrero J.J., Carter A., Carvalho F., Casey D.C., Caso V., Castañeda-Orjuela C.A., Castle C.D., Catalá-López F., Chang H.Y., Chang J.C., Charlson F.J., Chen H., Chibalabala M., Chibueze C.E., Chisumpa V.H., Chitheer A.A., Christopher D.J., Ciobanu L.G., Cirillo M., Colombara D., Cooper C., Cortesi P.A., Criqui M.H., Crump J.A., Dadi A.F., Dalal K., Dandona L., Dandona R., Das Neves J., Davitoiu D.V., De Courten B., De Leo D., Degenhardt L., Deiparine S., Dellavalle R.P., Deribe K., Des Jarlais D.C., Dey S., Dharmaratne S.D., Dhillon P.K., Dicker D., Ding E.L., Djalalinia S., Do H.P., Dorsey E.R., Dos Santos K.P.B., Douwes-Schultz D., Doyle K.E., Driscoll T.R., Dubey M., Duncan B.B., El-Khatib Z.Z., Ellerstrand J., Enayati A., Endries A.Y., Ermakov S.P., Erskine H.E., Eshrati B., Eskandarieh S., Esteghamati A., Estep K., Fanuel F.B.B., Farinha C.S.E.S., Faro A., Farzadfar F., Fazeli M.S., Feigin V.L., Fereshtehnejad S.M., Fernandes J.C., Ferrari A.J., Feyissa T.R., Filip I., Fischer F., Fitzmaurice C., Flaxman A.D., Flor L.S., Foigt N., Foreman K.J., Franklin R.C., Fullman N., Fürst T., Furtado J.M., Futran N.D., Gakidou E., Ganji M., Garcia-Basteiro A.L., Gebre T., Gebrehiwot T.T., Geleto A., Gemechu B.L., Gesesew H.A., Gething P.W., Ghajar A., Gibney K.B., Gill P.S., Gillum R.F., Ginawi I.A.M., Giref A.Z., Gishu M.D., Giussani G., Godwin W.W., Gold A.L., Goldberg E.M., Gona P.N., Goodridge A., Gopalani S.V., Goto A., Goulart A.C., Griswold M., Gugnani H.C., Gupta R., Gupta R., Gupta T., Gupta V., Hafezi-Nejad N., Hailu A.D., Hailu G.B., Hamadeh R.R., Hamidi S., Handal A.J., Hankey G.J., Hao Y., Harb H.L., Hareri H.A., Haro J.M., Harvey J., Hassanvand M.S., Havmoeller R., Hawley C., Hay R.J., Hay S.I., Henry N.J., Heredia-Pi I.B., Heydarpour P., Hoek H.W., Hoffman H.J., Horita N., Hosgood H.D., Hostiuc S., Hotez P.J., Hoy D.G., Htet A.S., Hu G., Huang H., Huynh C., Iburg K.M., Igumbor E.U., Ikeda C., Irvine C.M.S., Jacobsen K.H., Jahanmehr N., Jakovljevic M.B., Jassal S.K., Javanbakht M., Jayaraman S.P., Jeemon P., Jensen P.N., Jha V., Jiang G., John D., Johnson C.O., Johnson S.C., Jonas J.B., Jürisson M., Kabir Z., Kadel R., Kahsay A., Kamal R., Kan H., Karam N.E., Karch A., Karema C.K., Kasaeian A., Kassa G.M., Kassaw N.A., Kassebaum N.J., Kastor A., Katikireddi S.V., Kaul A., Kawakami N., Keiyoro P.N., Kengne A.P., Keren A., Khader Y.S., Khalil I.A., Khan E.A., Khang Y.H., Khosravi A., Khubchandani J., Kieling C., Kim D., Kim P., Kim Y.J., Kimokoti R.W., Kinfu Y., Kisa A., Kissimova-Skarbek K.A., Kivimaki M., Knudsen A.K., Kokubo Y., Kolte D., Kopec J.A., Kosen S., Koul P.A., Koyanagi A., Kravchenko M., Krishnaswami S., Krohn K.J., Kuate Defo B., Kucuk Bicer B., Kumar G.A., Kumar P., Kumar S., Kyu H.H., Lal D.K., Lalloo R., Lambert N., Lan Q., Larsson A., Lavados P.M., Leasher J.L., Lee J.T., Lee P.H., Leigh J., Leshargie C.T., Leung J., Leung R., Levi M., Li Y., Li Y., Li Kappe D., Liang X., Liben M.L., Lim S.S., Linn S., Liu A., Liu P.Y., Liu S., Liu Y., Lodha R., Logroscino G., London S.J., Looker K.J., Lopez A.D., Lorkowski S., Lotufo P.A., Low N., Lozano R., Lucas T.C.D., Macarayan E.R.K., Magdy Abd El Razek H., Magdy Abd El Razek M., Mahdavi M., Majdan M., Majdzadeh R., Majeed A., Malekzadeh R., Malhotra R., Malta D.C., Mamun A.A., Manguerra H., Manhertz T., Mantilla A., Mantovani L.G., Mapoma C.C., Marczak L.B., Martinez-Raga J., Martins-Melo F.R., Martopullo I., März W., Mathur M.R., Mazidi M., McAlinden C., McGaughey M., McGrath J.J., McKee M., McNellan C., Mehata S., Mehndiratta M.M., Mekonnen T.C., Memiah P., Memish Z.A., Mendoza W., Mengistie M.A., Mengistu D.T., Mensah G.A., Meretoja A., Meretoja T.J., Mezgebe H.B., Micha R., Millear A., Miller T.R., Mills E.J., Mirarefin M., Mirrakhimov E.M., Misganaw A., Mishra S.R., Mitchell P.B., Mohammad K.A., Mohammadi A., Mohammed K.E., Mohammed S., Mohanty S.K., Mokdad A.H., Mollenkopf S.K., Monasta L., Hernandez J.M., Montico M., Moradi-Lakeh M., Moraga P., Mori R., Morozoff C., Morrison S.D., Moses M., Mountjoy-Venning C., Mruts K.B., Mueller U.O., Muller K., Murdoch M.E., Murthy G.V.S., Musa K.I., Nachega J.B., Nagel G., Naghavi M., Naheed A., Naidoo K.S., Naldi L., Nangia V., Natarajan G., Negasa D.E., Negoi I., Negoi R.I., Newton C.R., Ngunjiri J.W., Nguyen C.T., Nguyen G., Nguyen M., Le Nguyen Q., Nguyen T.H., Nichols E., Ningrum D.N.A., Nolte S., Nong V.M., Norrving B., Noubiap J.J.N., O’Donnell M.J., Ogbo F.A., Oh I.H., Okoro A., Oladimeji O., Olagunju A.T., Olagunju T.O., Olsen H.E., Olusanya B.O., Olusanya J.O., Ong K., Opio J.N., Oren E., Ortiz A., Osgood-Zimmerman A., Osman M., Owolabi M.O., Pa M., Pacella R.E., Pana A., Panda B.K., Papachristou C., Park E.K., Parry C.D., Parsaeian M., Patten S.B., Patton G.C., Paulson K., Pearce N., Pereira D.M., Perico N., Pesudovs K., Peterson C.B., Petzold M., Phillips M.R., Pigott D.M., Pillay J.D., Pinho C., Plass D., Pletcher M.A., Popova S., Poulton R.G., Pourmalek F., Prabhakaran D., Prasad N., Prasad N.M., Purcell C., Qorbani M., Quansah R., Rabiee R.H.S., Radfar A., Rafay A., Rahimi K., Rahimi-Movaghar A., Rahimi-Movaghar V., Rahman M., Rahman M.H.U., Rai R.K., Rajsic S., Ram U., Ranabhat C.L., Rankin Z., Rao P.V., Rao P.C., Rawaf S., Ray S.E., Reiner R.C., Reinig N., Reitsma M.B., Remuzzi G., Renzaho A.M.N., Resnikoff S., Rezaei S., Ribeiro A.L., Ronfani L., Roshandel G., Roth G.A., Roy A., Rubagotti E., Ruhago G.M., Saadat S., Sadat N., Safdarian M., Safi S., Safiri S., Sagar R., Sahathevan R., Salama J., Salomon J.A., Salvi S.S., Samy A.M., Sanabria J.R., Santomauro D., Santos I.S., Santos J.V., Santric Milicevic M.M., Sartorius B., Satpathy M., Sawhney M., Saxena S., Schmidt M.I., Schneider I.J.C., Schöttker B., Schwebel D.C., Schwendicke F., Seedat S., Sepanlou S.G., Servan-Mori E.E., Setegn T., Shackelford K.A., Shaheen A., Shaikh M.A., Shamsipour M., Shariful Islam S.M., Sharma J., Sharma R., She J., Shi P., Shields C., Shigematsu M., Shinohara Y., Shiri R., Shirkoohi R., Shirude S., Shishani K., Shrime M.G., Sibai A.M., Sigfusdottir I.D., Silva D.A.S., Silva J.P., Silveira D.G.A., Singh J.A., Singh N.P., Sinha D.N., Skiadaresi E., Skirbekk V., Slepak E.L., Sligar A., Smith D.L., Smith M., Sobaih B.H.A., Sobngwi E., Sorensen R.J.D., Sousa T.C.M., Sposato L.A., Sreeramareddy C.T., Srinivasan V., Stanaway J.D., Stathopoulou V., Steel N., Stein D.J., Stein M.B., Steiner C., Steiner T.J., Steinke S., Stokes M.A., Stovner L.J., Strub B., Subart M., Sufiyan M.B., Suliankatchi Abdulkader R., Sunguya B.F., Sur P.J., Swaminathan S., Sykes B.L., Sylte D.O., Tabarés-Seisdedos R., Taffere G.R., Takala J.S., Tandon N., Tavakkoli M., Taveira N., Taylor H.R., Tehrani-Banihashemi A., Tekelab T., Temam Shifa G., Terkawi A.S., Tesfaye D.J., Tesssema B., Thamsuwan O., Thomas K.E., Thrift A.G., Tiruye T.Y., Tobe-Gai R., Tollanes M.C., Tonelli M., Topor-Madry R., Tortajada M., Touvier M., Tran B.X., Tripathi S., Troeger C., Truelsen T., Tsoi D., Tuem K.B., Tuzcu E.M., Tyrovolas S., Ukwaja K.N., Undurraga E.A., Uneke C.J., Updike R., Uthman O.A., Uzochukwu B.S.C., Van Boven J.F.M., Varughese S., Vasankari T., Venkatesh S., Venketasubramanian N., Vidavalur R., Violante F.S., Vladimirov S.K., Vlassov V.V., Vollset S.E., Wadilo F., Wakayo T., Wang Y.P., Weaver M., Weichenthal S., Weiderpass E., Weintraub R.G., Werdecker A., Westerman R., Whiteford H.A., Wijeratne T., Wiysonge C.S., Wolfe C.D.A., Woodbrook R., Woolf A.D., Workicho A., Wulf Hanson S., Xavier D., Xu G., Yadgir S., Yaghoubi M., Yakob B., Yan L.L., Yano Y., Ye P., Yimam H.H., Yip P., Yonemoto N., Yoon S.J., Yotebieng M., Younis M.Z., Zaidi Z., Zaki M.E.S., Zegeye E.A., Zenebe Z.M., Zhang X., Zhou M., Zipkin B., Zodpey S., Zuhlke L.J., Murray C.J.L. (2017). Global, regional, and national incidence, prevalence, and years lived with disability for 328 diseases and injuries for 195 countries, 1990–2016: A systematic analysis for the Global Burden of Disease Study 2016. Lancet.

[bb0170] Warming L., Hassager C., Christiansen C. (2002). Changes in bone mineral density with age in men and women: a longitudinal study. Osteoporos. Int..

[bb0175] Watson S.L., Weeks B.K., Weis L.J., Harding A.T., Horan S.A., Beck B.R. (2018). High-intensity resistance and impact training improves bone mineral density and physical function in postmenopausal women with osteopenia and osteoporosis: the LIFTMOR randomized controlled trial. J. Bone Miner. Res..

[bb0180] Zitzmann A.L., Shojaa M., Kast S., Kohl M., von Stengel S., Borucki D., Gosch M., Jakob F., Kerschan-Schindl K., Kladny B., Lange U., Middeldorf S., Peters S., Schoene D., Sieber C., Thomasius F., Uder M., Kemmler W. (2022). The effect of different training frequency on bone mineral density in older adults. A comparative systematic review and meta-analysis. Bone.

