# Supplementary material for: A comparison of different exercise intensities for improving bone mineral density in postmenopausal women with osteoporosis: A systematic review and meta-analysis
Source: Bone Rep. 2022 Oct 21;17:101631. doi: 10.1016/j.bonr.2022.101631 (PMC9615132; doi:10.1016/j.bonr.2022.101631)
Supplement: Supplementary file 1 — Literature search strategies [file mmc1.docx]

**Appendix 1: the Cochrane Central Register of Controlled　Trials (CENTRAL) search strategy**

**Participant Keywords:** (MeSH descriptor: [Menopause] explode all trees) AND (female):ti,ab,kw

**Intervention Keywords:** (MeSH descriptor: [Exercise] explode all trees) OR (MeSH descriptor: [Sports] explode all trees) OR (MeSH descriptor: [Physical Fitness] explode all trees) OR "physical activity"

**Study design Keywords:** ("randomized controlled trial" OR "controlled clinical trial" OR "randomised" OR "randomized" OR "placebo" OR "randomly" OR "trial")

**Appendix 2: PubMed search strategy**

**Participant Keywords:** ("female"[MeSH Terms] OR "female"[Title/Abstract]) AND ("menopause"[MeSH Terms] OR "menopause"[Title/Abstract])

**Intervention Keywords:** "exercise"[MeSH Terms] OR "exercise"[Title/Abstract] OR "exercise therapy"[MeSH Terms] OR "exercise therapy"[Title/Abstract] OR "sports"[MeSH Terms] OR "sports"[Title/Abstract] OR "physical fitness"[MeSH Terms] OR "physical fitness"[Title/Abstract] OR "physical activity"[Title/Abstract]

**Study design Keywords:** ("randomized controlled trial" [tiab]) OR ("controlled clinical trial"[tiab]) OR (randomised[tiab]) OR (randomized[tiab]) OR (placebo[tiab]) OR (randomly) OR (trial[ti])

"randomized controlled trial"[Title/Abstract] OR "controlled clinical trial"[Title/Abstract] OR "randomised"[Title/Abstract] OR "randomized"[Title/Abstract] OR "placebo"[Title/Abstract] OR "randomly"[All Fields] OR "trial"[Title]

**Appendix 3: CINAHL search strategy**

**Participant Keywords:** (MM "Menopause+") OR (MM "Postmenopause") OR TX postmenopaus* OR TX menopaus*

**Intervention Keywords:** (exercise OR sports OR “physical fitness” OR "physical activity")

**Study design Keywords:** ("randomized controlled trial" OR "controlled clinical trial" OR "randomised" OR "randomized" OR "placebo" OR "randomly" OR "trial")

**Appendix 4: Web of Science search strategy**

**Participant Keywords:** (female AND menopause)

**Intervention Keywords:** (exercise OR sports OR “physical fitness” OR "physical activity")

**Study design Keywords:** ("randomized controlled trial" OR "controlled clinical trial" OR "randomised" OR "randomized" OR "placebo" OR "randomly" OR "trial")

**Appendix 5: EMBASE search strategy**

**Participant Keywords:** ﻿(osteoporosis/ OR osteoporo* OR osteopenia* OR bone density/ OR bone mass/ OR bone densit* OR bone loss* OR bone mass* OR bone mineral/ OR bone mineral densit* OR bone mineral content*) AND (postmenopause/ OR postmenopause osteoporosis/ OR menopause/ OR (menopaus* OR postmenopaus* OR post-menopaus* OR (post NEAR menopause*)))

**Intervention Keywords:** (exp exercise/ OR exp sport/ OR fitness/ OR exp physical activity/ OR sport* OR exercis* OR physical fitness* OR physical activit* OR vibration therapy/ OR vibration therap*)

**Study design Keywords:** (randomized controlled trial/ OR double blind procedure/ OR single blind procedure/ OR triple Blind procedure/ OR randomi*: tiab OR randomisation/ OR placebo/ OR placebo* OR ((controlled OR comparative OR placebo OR random*) NEAR/3 (trial OR study))

(random*NEAR/7 (allocate*OR allot*OR assign*OR basis*OR divid*or order*))

((singl*OR doubl*OR trebl*OR tripl*) NEAR/3 (blind*OR mask*)))

**Appendix 6: MEDLINE search strategy**

**Participant Keywords:** ﻿(osteoporosis/ OR ﻿ osteoporo* OR osteopenia OR bone densit* OR bone OR bones OR bone loss* OR bone mass* OR bone mineral densit* OR bone mineral content* OR bone age OR bone defect* OR bone deminerali?ation OR bone mineral* OR bone strength OR decalcifi* OR deminerali?ed bone) AND (exp menopause/ OR (menopaus*OR postmenopaus*OR post-menopaus*OR (post adj menopause*)))

**Intervention Keywords:** (exp exercise/ OR exp exercise therapy/ OR exerci* OR exp sports/ OR sport* OR physical fitness OR physical activit* OR vibration/tu OR vibration therap*)

**Study design Keywords:** (randomized controlled trial OR controlled clinical trial OR randomi?ed:tiab OR placebo:ab OR clinical trials as topic OR randomly:tiab OR trial:ti NOT (animals not (humans and animals))
